# [RETRACTED ARTICLE]: EVALUATION OF ENEMAS CONTAINING SUCRALFATE IN TISSUE CONTENT OF MUC-2 PROTEIN IN EXPERIMENTAL MODEL OF DIVERSION COLITIS

**DOI:** 10.1590/0102-672020180001e1396

**Published:** 2018-12-10

ABCD staff team communicates formal Retraction about the article below:

Fernandez OOA, Pereira JA, Campos FG, Araya CM, Marinho GE, Novo RS, Oliveira TS, Franceschi YT, Martinez CAR. EVALUATION OF ENEMAS CONTAINING SUCRALFATE IN TISSUE CONTENT OF MUC-2 PROTEIN IN EXPERIMENTAL MODEL OF DIVERSION COLITIS. Arq Bras Cir Dig. 2018 Aug 16;31(3):e1391. doi: 10.1590/0102-672020180001e1391

since it was proved it's duplicate publication that was available in a previous edition of Brazilian Journal of Digestive Surgery

Fernandez OOA, Pereira JA, Campos FG, Araya CM, Marinho GE, Novo RS, Oliveira TS, Franceschi YT, Martinez CAR. EVALUATION OF ENEMAS CONTAINING SUCRALFATE IN TISSUE CONTENT OF MUC-2 PROTEIN IN EXPERIMENTAL MODEL OF DIVERSION COLITIS. Arq Bras Cir Dig. 2017 Apr-Jun;30(2):132-138. doi: 10.1590/0102-6720201700020012

Prof. Dr. Osvaldo Malafaia

Editor-in-Chief 

